# The effect of acute exercise on circulating immune cells in newly diagnosed breast cancer patients

**DOI:** 10.1038/s41598-023-33432-4

**Published:** 2023-04-21

**Authors:** Tiia Koivula, Salla Lempiäinen, Petteri Rinne, Jenna H. Rannikko, Maija Hollmén, Carl Johan Sundberg, Helene Rundqvist, Heikki Minn, Ilkka Heinonen

**Affiliations:** 1 Turku PET Centre, University of Turku and Turku University Hospital, Kiinamyllynkatu 4-8, 20520 Turku, Finland; 2 Institute of Biomedicine, University of Turku, Turku, Finland; 3 MediCity Research Laboratory, University of Turku, Turku, Finland; 4 Department of Physiology and Pharmacology, Karolinska Institutet, Stockholm, Sweden; 5 Department of Learning, Informatics, Management and Ethics, Karolinska Institutet, Stockholm, Sweden; 6 Department of Laboratory Medicine, Karolinska Institutet, Stockholm, Sweden; 7 Department of Oncology and Radiotherapy, Turku University Hospital, Turku, Finland; 8 Rydberg Laboratory of Applied Sciences, University of Halmstad, Halmstad, Sweden

**Keywords:** Cancer, Immunology

## Abstract

The role of exercise in cancer prevention and control is increasingly recognized, and based on preclinical studies, it is hypothesized that mobilization of leukocytes plays an important role in the anti-tumor effect. Thus, we examined how 10-min acute exercise modulates immune cells in newly diagnosed breast cancer patients. Blood samples were taken at rest, immediately after exercise and 30 min after exercise and phenotypic characterization of major leukocyte subsets was done using 9-color flow cytometry. Total leukocyte count increased by 29%, CD8^+^ T cell count by 34%, CD19^+^ B cell count by 18%, CD56^+^CD16^+^ NK cell count by 130%, and CD14^+^CD16^+^ monocyte count by 51% immediately after acute exercise. Mobilization of CD45^+^, CD8^+^, CD19^+^, and CD56^+^CD16^+^ cells correlated positively with exercising systolic blood pressure, heart rate percentage of age predicted maximal heart rate, rate pressure product, and mean arterial pressure. Our findings indicate that a single bout of acute exercise of only 10 min can cause leukocytosis in breast cancer patients. Mobilization of leukocytes appear to be directly related to the intensity of exercise. It is possible that the positive effect of exercise on oncologic outcome might be partly due to immune cell mobilization as documented in the present study.

## Introduction

Breast cancer is the most commonly diagnosed cancer among women worldwide^1^. The increasing incidence rate of breast cancer has been attributed to multiple factors including enhanced screening, an ageing population, but also lifestyle related factors such as dietary changes, obesity, and lack of exercise^2^. On the other hand, post-diagnosis physical activity has been associated with better quality of life including physical, functional, and psychological well-being^3^. In epidemiological studies, exercise has also been associated with better outcome in breast cancer patients. Holick et al.^4^ reported that weekly exercise of moderate intensity (5 METs) reduced the mortality rate by 15%. Holmes et al.^5^ also reported that the mortality risk reduction was 6% for women who engaged in ≥ 9 MET-h/ wk compared to ≤ 3 MET-h/ wk. This positive effect of exercise is most likely mediated by several different mechanisms, one of which is through immunomodulation^6^. The capacity of the immune system to combat cancer has been known for several decades^7^. CD8^+^ cytotoxic T cells, gamma delta T cells, and natural killer (NK) cells are primarily responsible for killing tumor cells, while other immune cells such as CD4^+^ helper T cells can enhance their cytotoxic effect^8,9^.

The effect of acute and regular exercise on immune cell count has been extensively investigated in healthy individuals^10–13^ and it is widely accepted that physical exercise can alter the number and function of leukocytes. Increased immune cell concentrations and activities following exercise bout are mediated by distinct mechanisms, for example by adrenergic system and muscle-derived IL-6^11,14^. However, it is widely accepted that findings in healthy individuals can not be generalized to diseased states, such as cancer. In breast cancer patients, the effect of exercise has been examined during and after cancer treatments^15,16^. However, to the best of our knowledge there are no studies investigating the effects of acute exercise on immune cell counts in breast cancer patients before the start of cancer treatments. Cancer and its treatments are associated with altered white blood cell count^17^ and that is why the effect of exercise on immune cells might vary depending on the health status and should thus be the focus of the research.

Consequently, the aim of this study was to examine the changes in immune cell counts after short acute exercise in newly diagnosed breast cancer patients before the start of cancer treatments.

## Results

### Baseline and exercise characteristic

Mean heart rate was 66 ± 9 bpm at rest and increased significantly to 114 ± 17 bpm during exercise (*p* < 0.001). A heart rate percentage from age predicted maximal heart rate was 70 ± 10% during exercise. Systolic blood pressure was 140 ± 30 mmHg at rest and increased significantly to 158 ± 30 mmHg during exercise (*p* < 0.001) and diastolic blood pressure was 75 ± 12 mmHg at rest and increased significantly to 89 ± 29 mmHg during exercise (*p* < 0.01). MAP was 96 ± 17 mmHg at rest and increased significantly to 112 ± 28 mmHg during exercise (*p* < 0.001). RPP was 9249 ± 2679 bpm mmHg at rest and increased significantly to 17,996 ± 4718 bpm mmHg during exercise (*p* < 0.001). Exercising power production ranged from 15 to 75 watts being on average 50 ± 19 watts. Strenuousness of the exercise was on average 12 ± 2 on Borg scale.

### Immune cell responses to acute exercise

**Figure 1:**
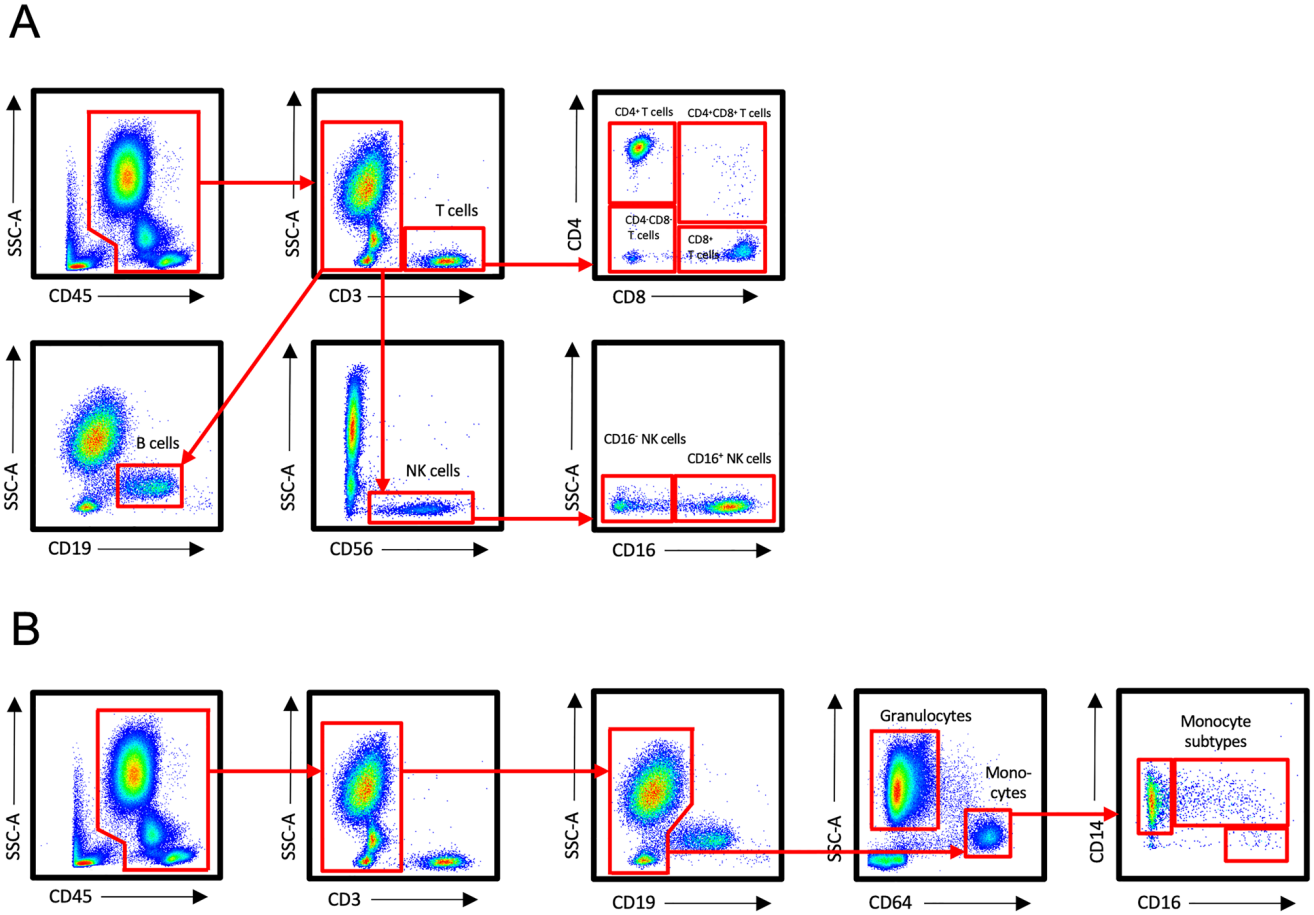
Flow cytometry gating strategy of immune cells studied. (**A**) staining panel 1, (**B**) staining panel 2.

Counts of total leukocytes, T cells, B cells, NK cells, granulocytes, and monocytes were examined in the peripheral blood of 20 breast cancer patients at rest, immediately after exercise, and 30 min after exercise with flow cytometry. Gating strategy is presented in Fig. 1.

**Figure 2:**
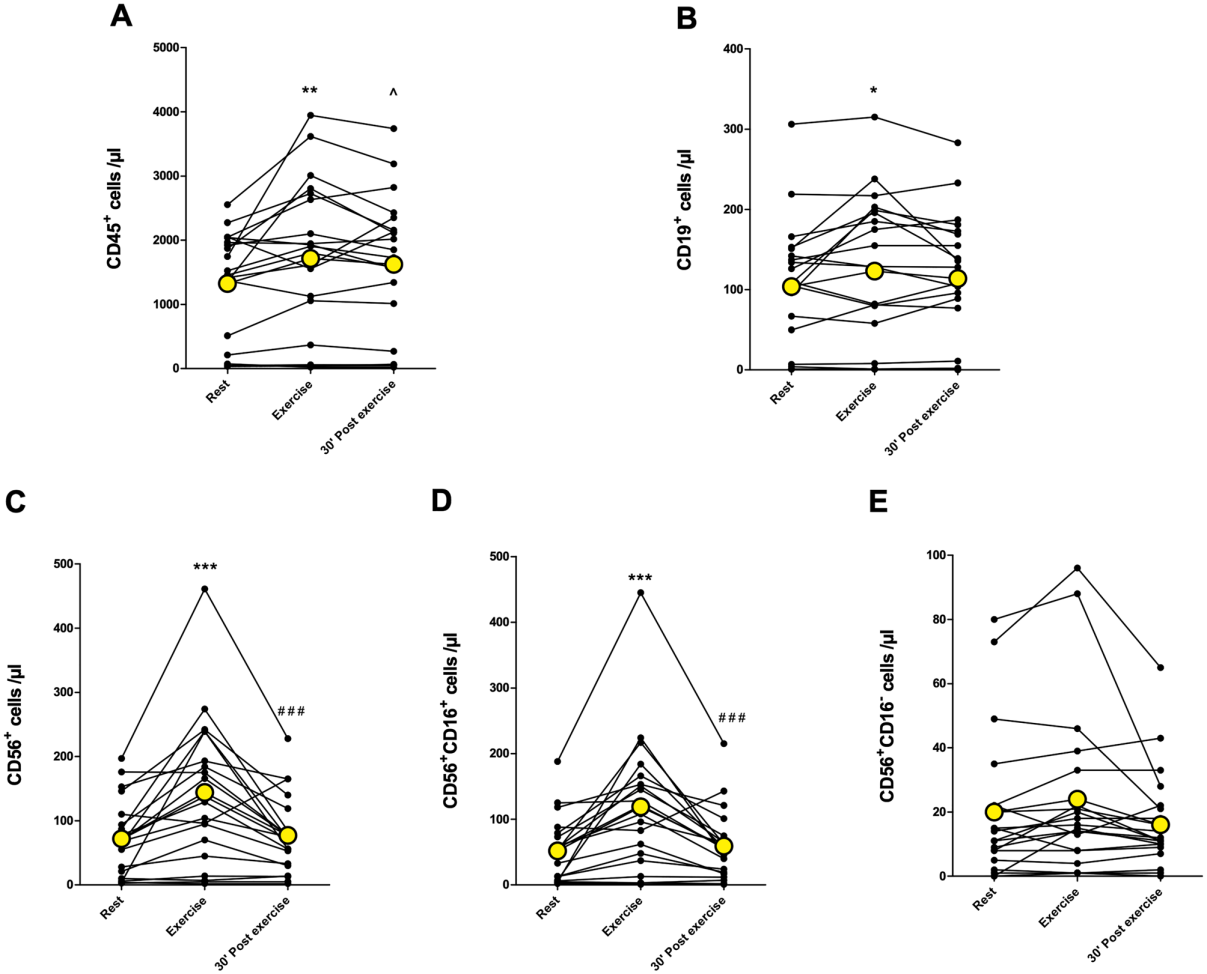
The number of total leukocytes, B cells, and NK cells at rest, immediately after and 30 min after acute exercise. (**A**) Leukocyte (CD45^+^) count increased significantly immediately after exercise and was above baseline after 30 min post-exercise. (**B**) The number of B cells (identified as CD45^+^CD3^−^CD19^+^ cells) increased significantly immediately after exercise. (**C**) Total NK cell (identified as CD45^+^CD3^−^CD56^+^ cells) and (**D**) CD56^+^CD16^+^ NK cell count increased significantly immediately after exercise and decreased to baseline levels at 30 min post-exercise. (**E**) Acute exercise had no significant effect on CD56^+^CD16^−^ NK cells. Yellow points represent the mean. *p < 0.05 between rest and exercise; **p < 0.01 between rest and exercise;***p < 0.001 between rest and exercise; ^*p* < 0.05 between rest and 30 min post-exercise; ###*p* < 0.001 exercise and 30 min post-exercise.

**Figure 3:**
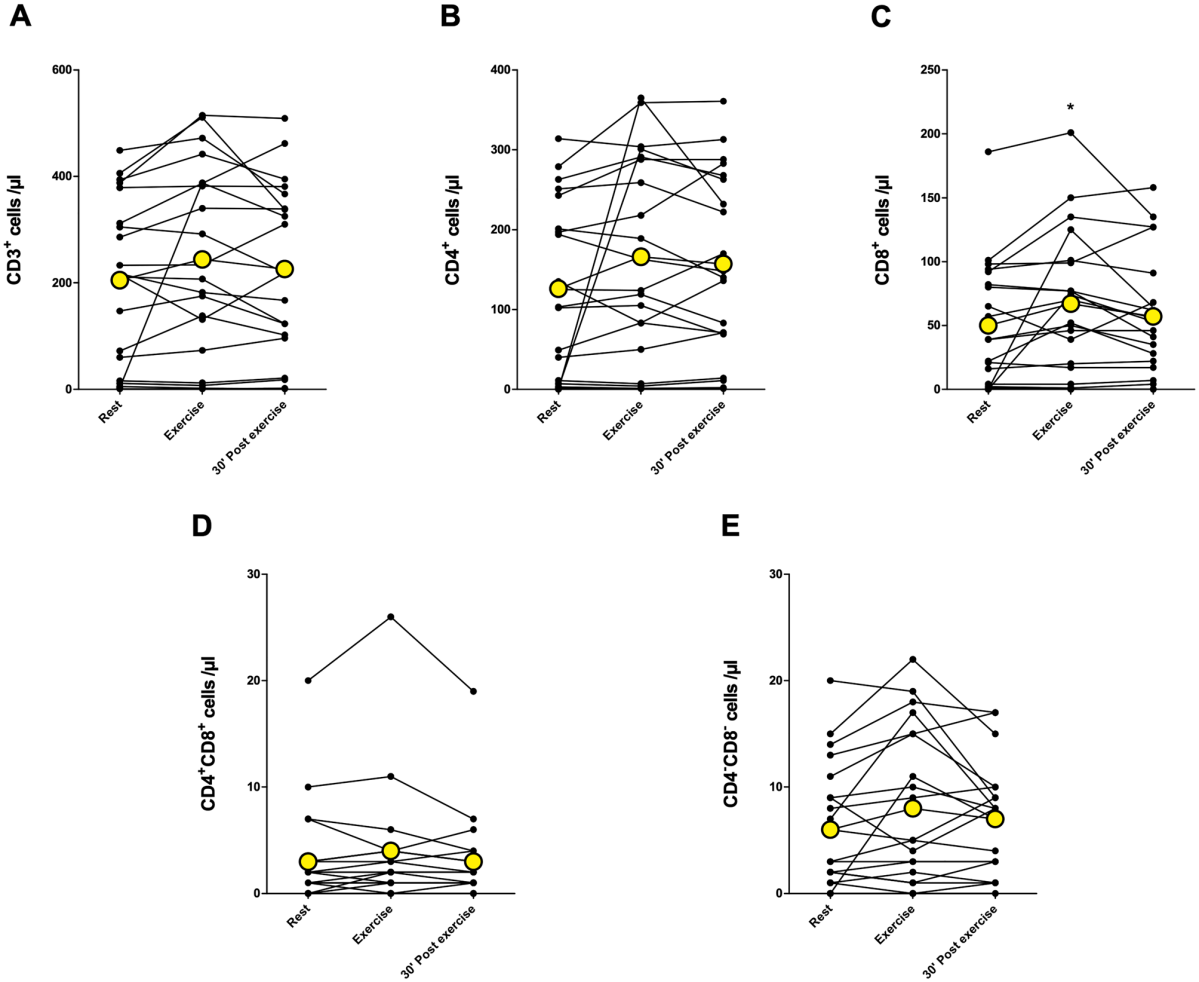
The number of T cells at rest, immediately after and 30 min after acute exercise. The number of (**A**) total T cells (CD45^+^CD3^+^) or the number of (**B**) helper T cells (identified as CD45^+^CD3^+^CD4^+^ cells) did not change. The number of (**C**) cytotoxic T cells (identified as CD45^+^CD3^+^CD8^+^ cells) increased significantly immediately after exercise. The number of (**D**) double positive T cells and the number of (**E**) double negative T cells (identified as CD45^+^CD3^+^CD4^−^CD8^−^ cells remained unchanged. Yellow points represent the mean. *p < 0.05 between rest and exercise.

**Figure 4:**
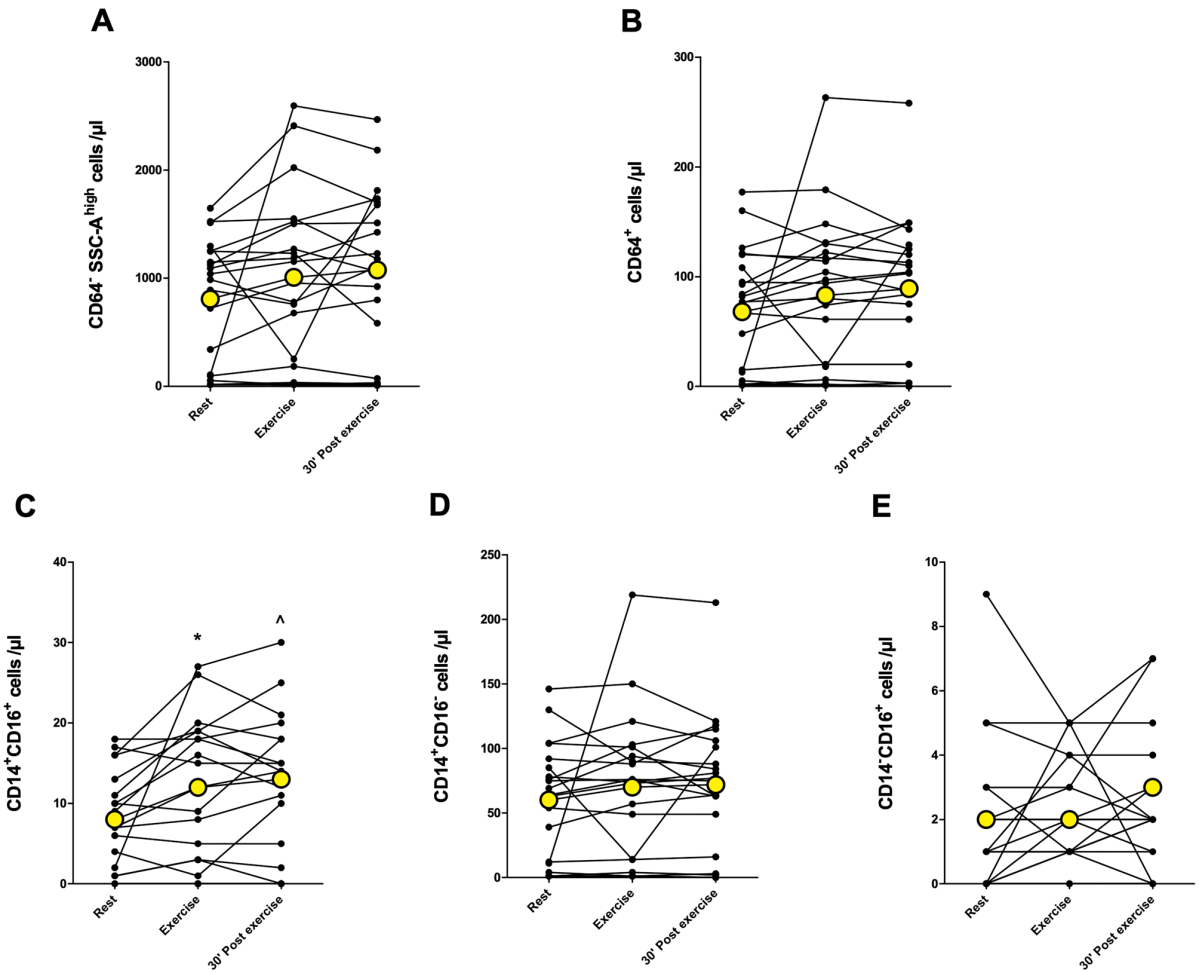
The number of granulocytes and monocytes at rest, immediately after and 30 min after acute exercise. The number of (**A**) granulocytes (identified as CD45^+^CD3^−^CD19^−^CD64^−^SSC-A^high^ cells) or (**B**) total monocytes (identified as CD45^+^CD3^−^CD19^−^CD64^+^ cells) did not change. (**C**) Acute exercise significantly increased intermediate monocyte count (identified as CD45^+^CD3^−^CD19^−^CD64^+^CD14^+^CD16^+^ cells) immediately after exercise and the levels were above baseline at 30 min post-exercise. There were no significant changes in (**D**) classical monocytes (identified as CD45^+^CD3^−^CD19^−^CD64^+^CD14^+^CD16^−^ cells) or (**E**) non-classical monocytes (identified as CD45^+^CD3^−^CD19^−^CD64^+^CD14^−^CD16^+^ cells). Yellow points represent the mean. *p < 0.05 between rest and exercise; ^*p* < 0.05 between rest and 30 min post-exercise.

The total number of leukocytes (CD45^+^) increased immediately after 10-min acute exercise (*p* < 0.01) and was above baseline levels after 30-min rest (*p* < 0.05) (Fig. 2A). The number of CD19^+^ B cells increased immediately after exercise (*p* < 0.05) and returned back to baseline at 30 min post-exercise (Fig. 2B). Similarly, the total number of NK cells and the number of CD56^+^CD16^+^ NK cells increased immediately after exercise (*p* < 0.001) and decreased back to baseline levels at 30 min post-exercise (Fig. 2C,D). There was no significant change in CD56^+^CD16^−^ NK cell count (Fig. 2E). The total number of T cells (CD3^+^) or the number of CD4^+^ helper T cells did not change after the exercise (Fig. 3A,B). The number of CD8^+^ cytotoxic T cells increased significantly immediately after exercise (*p* < 0.05) and returned to baseline at 30 min post-exercise (Fig. 3C). Further, there was no change in CD4^+^CD8^+^ double positive T cells or CD4^−^ CD8^−^ double negative T cells (Fig. 3D,E). The number of granulocytes and total monocytes did not change in response to exercise (Fig. 4A,B). However, the number of CD14^+^CD16^+^ monocytes increased immediately after exercise (*p* < 0.05) and was significantly above baseline at 30 min post-exercise (*p* < 0.05) (Fig. 4C). There was no significant change in the number of classical (CD14^+^CD16^−^) or non-classical (CD14^−^CD16^+^) monocytes (Fig. 4D,E).

The proportions of the immune cells in response to exercise are presented in Table 1. The percentage of total NK cells and CD56^+^CD16^+^ NK cells of total leukocytes increased immediately after exercise (*p* < 0.01 and *p* < 0.001, respectively), and decreased back to baseline at 30 min post-exercise. The percentage of CD8^+^ T cells of total T cells increased immediately after exercise (*p* < 0.05). Moreover, the percentage of CD14^+^CD16^+^ monocytes of total monocytes increased immediately after exercise (*p* < 0.01), and remained elevated at 30 min post-exercise (*p* < 0.05). The proportions of other studied immune cells did not change significantly (Table 1).

**Table 1:** Proportional count of immune cells in response to acute exercise.

	Rest	Exercise	30 min post-exercise
% of total leukocytes (CD45^+^)			
CD3^+^	16.57 (9.5)	14.23 (6.20)	15.72 (9.23)
CD4^+^	10.45 (6.93)	9.38 (3.88)	10.83 (6.29)
CD8^+^	4.00 (3.00)	4.06 (2.74)	3.96 (2.84)
CD4^+^CD8^+^	0.27 (0.30)	0.21 (0.18)	0.20 (0.21)
CD4^−^CD8^−^	0.76 (1.17)	0.57 (0.52)	0.63 (0.94)
CD19^+^	6.90 (2.82)	6.30 (2.87)	6.15 (2.34)
CD56^+^	7.25 (5.06)	10.17 (6.48)**	7.42 (6.71)^#^
CD56^+^CD16^+^	4.89 (3.60)	8.26 (6.45)***	5.47 (5.32)^##^
CD56^+^CD16^−^	2.33 (2.60)	1.87 (1.53)	1.91 (2.55)
CD64^−^ SSC-A^high^	58.41 (18.93)	56.49 (16.35)	60.33 (20.26)
CD64^+^	5.54 (2.01)	5.19 (1.74)	5.29 (1.93)
CD14^+^CD16^+^	0.53 (0.36)	0.61 (0.31)	0.59 (0.41)
CD14^+^CD16^−^	4.57 (1.64)	4.15 (1.36)	4.19 (1.47)
CD14^−^CD16^+^	0.16 (0.12)	0.18 (0.19)	0.15 (0.15)
% of total T cells (CD3^+^)			
CD4^+^	65.73 (18.80)	67.80 (11.42)	69.79 (9.62)
CD8^+^	23.20 (11.11)	26.33 (10.26)*	24.62 (8.52)
CD4^+^CD8^+^	1.75 (1.67)	1.69 (1.72)	1.54 (1.54)
CD4^−^CD8^−^	4.35 (4.65)	4.15 (4.12)	3.54 (2.79)
% of total monocytes (CD64^+^)			
CD14^+^CD16^+^	9.05 (4.33)	11.45 (5.05)**	11.24 (5.56)^
CD14^+^CD16^−^	82.84 (5.34)	80.24 (6.23)	80.33 (9.03)
CD14^−^CD16^+^	2.87 (2.25)	3.12 (2.56)	2.97 (1.97)

Data presented as mean (SD).

Significant *p* values; * < 0.05 between rest and exercise; ** < 0.01 between rest and exercise; *** < 0.001 between rest and exercise; ^ < 0.05 between rest and 30 min post-exercise; ^#^ < 0.05 between exercise and 30 min post-exercise; ^##^ < 0.01 between exercise and 30 min post-exercise.

### The correlation between exercise intensity and immune cell mobilization

Correlations between exercise intensity variables and immune cell mobilization are shown in Table 2. Change in CD45^+^, CD3^+^, CD4^+^, CD8^+^, CD4^+^CD8^+^, CD4^−^CD8^−^, CD19^+^, CD56^+^, CD56^+^CD16^+^, and CD56^+^CD16^−^ cell counts between rest and exercise correlated positively with exercising systolic blood pressure and rate pressure product (*p* < 0.05 in all correlations). Change in CD45^+^, CD3^+^, CD4^+^, CD8^+^, CD4^−^CD8^−^, CD19^+^, CD56^+^, and CD56^+^CD16^−^ cell counts correlated positively with exercising diastolic blood pressure (*p* < 0.05 in all correlations). Change in CD45^+^, CD4^−^CD8^−^, CD56^+^, and CD56^+^CD16^+^ cell counts correlated positively with heart rate during acute exercise (*p* < 0.05 in all correlations), and change in CD45^+^, CD3^+^, CD8^+^, CD4^−^CD8^−^, CD19^+^, CD56^+^, and CD56^+^CD16^+^ cell counts correlated positively with heart rate percentage of age predicted maximal heart rate (*p* < 0.05 in all correlations). Further, exercising mean arterial pressure correlated positively with change in CD45^+^, CD3^+^, CD4^+^, CD8^+^, CD4^-^CD8^−^, CD19^+^, CD56^+^, CD56^+^CD16^+^, and CD56^+^CD16^−^ cell counts (*p* < 0.05 in all correlations). There were no significant associations between immune cell mobilization and pedaling power (watts). Age correlated positively only with change in CD4^+^CD8^+^ cell count and BMI correlated positively with resting heart rate but had no effect on immune cell mobilization.

**Table 2:** Pearson correlation between age, BMI, and exercise intensity variables and change in the cell number between rest and exercise.

	Age, years	BMI, kg/m^2^	SBP, mmHg	DBP, mmHg	HR, bpm	HR% of HRmax	RPP, bpm*mmHg	MAP, mmHg	Pedaling power, watts
ΔCD45^+^	0.1907	0.1505	**0**.6514**	**0**.5552*	**0**.4674*	**0**.5823**	**0**.7027***	**0**.5830**	− 0.1473
ΔCD3^+^	0.1939	0.3843	**0**.7542***	**0**.8779***	0.3745	**0**.4838*	**0**.8056***	**0**.8629***	− 0.2111
ΔCD4^+^	0.2184	0.2187	**0**.6040**	**0**.7279***	0.3098	0.4352	**0**.6534**	**0**.7057***	− 0.0416
ΔCD8^+^	0.0724	0.2788	0.4887*	0.5815**	0.4432	0.4903*	0.6239**	0.5646**	0.0447
ΔCD4^+^CD8^+^	0.4701*	− 0.0323	0.5035*	0.3917	0.1917	0.4259	0.5050*	0.4455	− 0.2411
ΔCD4^−^CD8^−^	0.1898	0.2929	0.6492**	0.6798***	0.4811*	0.5926**	0.7693***	0.6902***	− 0.0115
ΔCD19^+^	0.2176	0.2389	0.5384*	0.5799**	0.4077	0.5389*	0.6410**	0.5873**	− 0.1826
ΔCD56^+^	0.1912	0.3363	0.4961*	0.5799*	0.5036*	0.6131**	0.6483**	0.4888*	0.0343
ΔCD56^+^CD16^+^	0.1828	0.3386	0.4695*	0.4356	0.4972*	0.6131**	0.6236**	0.4623*	0.0529
ΔCD56^+^CD16^−^	0.1868	0.1558	0.5299*	0.5001*	0.3156	0.4078	0.5846**	0.5275*	− 0.1794
ΔCD64^−^ SSC-A^high^	0.2386	− 0.0447	0.3163	0.2498	0.2664	0.3926	0.3853	0.2832	− 0.1882
ΔCD64^+^	0.3540	− 0.2558	0.2804	0.1860	0.0802	0.2667	0.2602	0.2282	− 0.2667
ΔCD14^+^CD16^+^	− 0.0287	− 0.0450	0.1223	0.0666	0.3432	0.3787	0.2709	0.0913	− 0.1077
ΔCD14^+^CD16^−^	0.3903	− 0.2903	0.2960	0.1924	0.0345	0.2379	0.2488	0.2382	− 0.2889
ΔCD14^−^CD16^+^	0.3938	− 0.2511	0.1080	0.1057	− 0.1693	0.0740	0.0067	0.1115	− 0.2971

Significant *p* values; * < 0.05, ** < 0.01, *** < 0.001.

*SBP* systolic blood pressure; *DBP* diastolic blood pressure; *HR* heart rate; *RPP* rate pressure product; *MAP* mean arterial pressure.

The correlations are calculated with exercising values of blood pressure, heart rate, RPP, and MAP.

Significant values are in [bold].

### The correlation between disease status and immune cell levels

Cancer grade correlated negatively with CD4^+^ count at baseline (r = − 0.4856, *p* = 0.0300). Lymph node involvement correlated negatively with total leukocyte count (r = − 0.4847, *p* = 0.0303), CD3^+^ T cell count (r = − 0.5433, *p* = 0.0135), CD4^+^ T cell count (r = − 0.4515, *p* = 0.0457), CD19^+^ B cell count (r = − 0.5274, *p* = 0.0169), and CD14^+^CD16^+^ monocyte count (r = − 0.5217, *p* = 0.0264) at baseline. Moreover, progesterone receptor positivity correlated negatively with total leukocyte count (r = − 0.4940, *p* = 0.0268), CD3^+^ T cell count (r = − 0.4856, *p* = 0.0300), CD8^+^ T cell count (r = − 0.5746, *p* = 0.0081), CD4^−^CD8^−^ T cell count (r = − 0.6285, *p* = 0.0030), and CD19^+^ B cell count (r = − 0.5029, *p* = 0.0238) at baseline. Correlations between disease status variables and immune cell mobilization by exercise were analysed in detail and are presented in Table 3. Estrogen receptor positivity correlated negatively with exercise-induced change in CD8^+^ T cells, and HER2 positivity correlated positively with change in CD4^+^ T cells and CD8^+^ T cells (*p* < 0.05 in all correlations). Grade, tumor size, lymph node involvement, metastasis, ductal or lobular subtype, progesterone receptor status, or proliferation index (Ki-67) did not correlate with exercise-induced immune cell mobilization.

**Table 3:** Pearson correlation between disease status and change in the cell number between rest and exercise.

	Grade, scale 1–3	Tumor size, scale 1–3	Lymph node involvement, scale 1–3	Metastasis, 0/1	Ductal/lobular subtype	ER, %	PR, %	HER2, pos/neg	Ki-67, %
ΔCD45^+^	0.1678	0.1697	− 0.0689	− 0.0598	− 0.0026	− 0.1279	− 0.3259	0.1841	0.0108
ΔCD3^+^	− 0.0054	0.2333	0.1123	− 0.0227	0.0856	− 0.1595	− 0.1867	0.1660	0.0991
ΔCD4^+^	0.1191	0.0179	− 0.0421	− 0.0744	− 0.0149	− 0.3522	− 0.0797	0.5246*	0.2143
ΔCD8^+^	0.1918	− 0.0517	− 0.1406	− 0.0182	− 0.1417	− 0.5409*	− 0.2709	0.6138**	0.2804
ΔCD4^+^CD8^+^	0.0841	0.0569	0.0103	0.0283	− 0.2097	0.1178	− 0.2730	0.0283	− 0.0507
ΔCD4^−^CD8^−^	0.1757	0.0478	− 0.1328	− 0.1274	− 0.0375	− 0.2782	− 0.2889	0.3243	0.1236
ΔCD19^+^	0.1884	0.3236	0.0284	− 0.0256	0.2169	− 0.0156	− 0.1824	0.1693	− 0.0826
ΔCD56^+^	− 0.1812	− 0.0513	− 0.2808	− 0.1786	− 0.0609	− 0.2280	− 0.1288	0.1935	− 0.1259
ΔCD56^+^CD16^+^	− 0.1949	− 0.0608	− 0.2962	− 0.2016	− 0.0308	− 0.2126	− 0.0962	0.1688	− 0.1446
ΔCD56^+^CD16^−^	0.0322	0.0797	0.0231	0.1418	− 0.3174	− 0.2508	− 0.3836	0.3313	0.1081
ΔCD64^−^ SSC-A^high^	0.1833	0.0932	− 0.0848	− 0.0174	− 0.1323	0.0571	− 0.2737	0.0840	− 0.0921
ΔCD64^+^	0.1456	0.0839	− 0.0236	− 0.0074	− 0.0405	0.2475	− 0.1969	0.0547	− 0.1550
ΔCD14^+^CD16^+^	0.3616	0.2259	− 0.0977	− 0.0781	− 0.0341	− 0.0781	− 0.3436	0.1023	0.0952
ΔCD14^+^CD16^−^	0.1226	0.0740	− 0.0058	− 0.0028	− 0.0465	0.2836	− 0.1816	0.0346	− 0.1594
ΔCD14^−^CD16^+^	0.1514	0.2115	0.1955	0.0421	0.2142	0.4406	0.1151	0.0638	− 0.3248

Significant *p* values; * < 0.05, ** < 0.01.

*ER* estrogen receptor; *PR* progesterone receptor; *HER2* human dermal growth factor receptor-2; *KI-67* proliferation index protein.

Significant values are in [bold].

## Discussion

**Figure 5:**
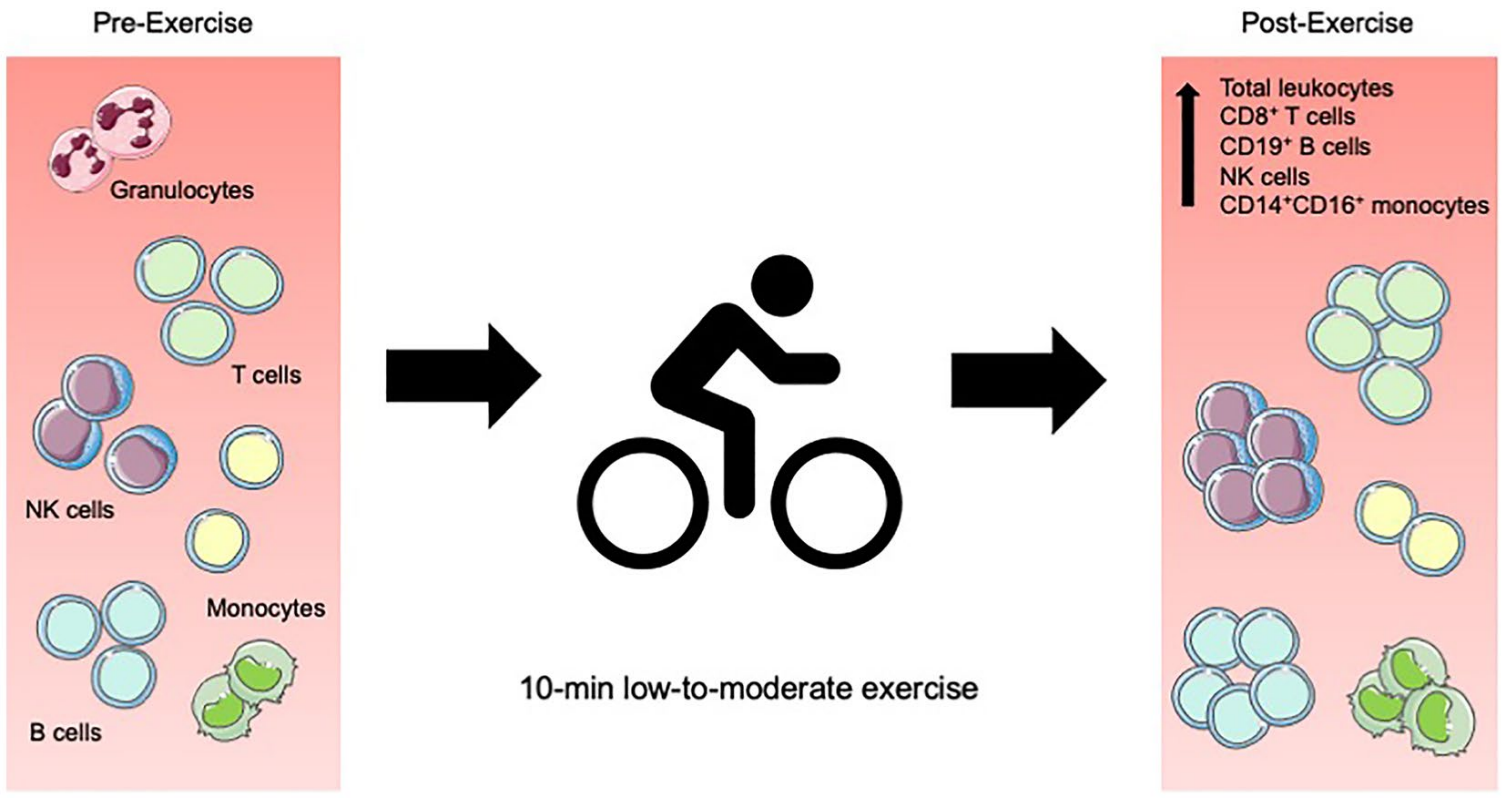
Acute exercise response in breast cancer patients. The number of several leukocyte subpopulations increase in blood following an acute exercise.

In the current study we examined the effect of acute exercise on blood immune cell counts in newly diagnosed breast cancer patients who had not yet started any treatments. We found that acute exercise led to a significant increase of total leukocyte (CD45^+^), CD8^+^ T cell, CD19^+^ B cell, total NK cell, CD56^+^CD16^+^ NK cell, and CD14^+^CD16^+^ monocyte counts in the bloodstream. The phenomenon is illustrated in Fig. 5. Furthermore, the proportion of total NK cells and CD56^+^CD16^+^ NK cell of total leukocytes, proportion of CD8^+^ T cells of total T cells, and proportion of CD14^+^CD16^+^ monocytes of total monocytes increased significantly with exercise. Mobilization of CD45^+^, CD8^+^, CD19^+^, and CD56^+^CD16^+^ cells correlated positively with exercising systolic blood pressure, heart rate percentage of age predicted maximal heart rate, rate pressure product, and mean arterial pressure. Moreover, mobilization of CD45^+^, CD8^+^, and CD19^+^ cells correlated positively with exercising diastolic blood pressure and mobilization of CD45^+^ and CD56^+^CD16^+^ cells correlated positively with heart rate during exercise.

Several studies, performed mostly in healthy volunteers, have documented that acute exercise is a powerful stimulus for mobilizing immune cells^18–21^. Acute exercise increases the concentration of several stress hormones in the blood, including epinephrine and norepinephrine and a correlation exists between the numbers of adrenergic receptors on leukocyte subpopulations and leukocyte responsiveness to exercise^22^. In the present clinical study, we found that the total leukocyte (CD45^+^) count increased by 29% immediately after acute exercise. The highest mobilization was detected for CD56^+^CD16^+^ NK cells, which is consistent with previously reported data as NK cells have been found to be the most responsive immune cells to acute exercise in healthy individuals^23^ and NK cells have the highest expression of adrenergic receptors among leukocytes^24^. Evans et al.^25^ have studied NK cell mobilization in response to 30-min acute exercise in breast cancer survivors and also found a significant increase in total NK cell number immediately post-exercise. We also found that CD8^+^ cytotoxic T cells and CD19^+^ B cells were mobilized during acute exercise, but the 34% and 18% increases were much lower compared to 130% in CD56^+^CD16^+^ NK cells. To the best of our knowledge, the effect of acute exercise on T and B lymphocytes has not been studied in breast cancer patients but recent studies with healthy individuals shows that acute exercise doubles the number of B cells^20^ and CD8^+^ T cells^26^ in the blood. Some breast tumors have tertiary lymphoid structures (TLS) which are characterized by dense follicles of B cells. According to the current understanding, B cells migrate to TLSs to induce anti-tumor responses^27,28^. To this date, however, we do not know whether the presence of TLSs has an effect on B cell response to acute exercise. In the present study we did not find significant changes in CD4^+^ T cells, as previously seen in healthy individuals^19,21^, which might be because of compromised immune cell function in these cancer patients. In addition, there was no change in CD4^+^CD8^+^ double positive T cells, CD4^−^CD8^−^ double negative T cells, or granulocytes. It might also be that higher exercise intensities and/or longer exercise duration are needed to show increases in these cells in cancer patients. Relatively high exercise intensities and durations have often been used in exercise studies in healthy volunteers. For example, Shek et al.^19^ used 65% of VO2_max_ for 120 min and Pistillo et al.^29^ used 80% of VO2_max_ for 30 min to find elevated T cell concentrations in healthy people. Interestingly, in this study we saw that change in total T cells, and CD4^+^, CD4^+^CD8^+^, and CD4^−^CD8^−^ T cells correlated positively with exercise intensity, even if there was no significant change in these T cell concentrations in response to exercise.

A recent study with breast cancer survivors showed that the number of intermediate (CD14^+^CD16^+^) and classical (CD14^+^CD16^−^) monocytes were increased after 45-min acute interval exercise but that the baseline resting levels of monocytes did not change after 16 weeks of chronic exercise training^15^. In the present study, we found that the amount of CD14^+^CD16^+^ monocytes in the bloodstream increased immediately following a 10-min acute exercise bout. The exercise intensity in our study is substantially lower compared to the above-mentioned study, which might explain why we did not find significant change in CD14^+^CD16^−^ monocytes and that the increase in CD14^+^CD16^+^ monocytes was lower in our study (51% in our study versus 121% in Khosravi et al.^15^. Moreover, it seems that chronic exercise does not change the immune cell counts in breast cancer patients^16^ or survivors^15^. The mobilization induced by acute exercise appears to be transient, as also noticed in the present study. In our study, the number of NK cells, cytotoxic T cells, and B cells decreased to baseline levels during 30-min supine rest. The total number of leukocytes (CD45^+^) and the number of CD14^+^CD16^+^ monocytes were however still significantly above baseline levels at 30 min post-exercise.

Additionally, exercise altered the proportions of some leukocyte subtypes in the blood. We found increases in percentage of total NK cells and CD56^+^CD16^+^ NK cells of total leukocytes, in the percentage of CD8^+^ T cells of total T cells, and in the percentage of CD14^+^CD16^+^ monocytes of total monocytes immediately after exercise. The percentages of NK cells and CD8^+^ T cells decreased back to baseline at 30 min post-exercise but the percentage of CD14^+^CD16^+^ monocytes remained elevated at 30 min post-exercise. Hence, the changes in cell proportions are in line with changes observed in absolute cell counts. Zimmer et al.^30^ have studied the effect of half marathon on immune cell proportions in breast cancer survivors and found increased proportion of granulocytes and decreased proportions of monocytes and lymphocytes after the run. Half marathon is however substantially more strenuous exercise bout compared to the one in the present study, and the patients have different health status, which might explain why our results partially contradict those of Zimmer et al. In healthy individuals, exercise has been found to increase the proportions of CD8^+^ T cells and NK cells^19,31^.

We observed an association between immune cell mobilization and exercise intensity. The increase in CD45^+^, CD8^+^, CD19^+^, CD56^+^, and CD56^+^CD16^+^ cell counts immediately after exercise correlated positively with heart rate percentage of age predicted maximal heart rate, thus with the surrogate of exercise intensity. Moreover, changes of several leukocyte subsets between rest and exercise correlated positively with exercising blood pressure, heart rate, rate pressure product, and mean arterial pressure. Previous studies have also reported that the effect of exercise might be dependent on the intensity, duration, and type of exercise. Schlagheck et al.^18^ showed that all immune cells except CD8^+^ T cells mobilized more after endurance training compared to resistance training. Da Silva Neves et al.^32^ found a greater increase in leukocytes after high-intensity exercise compared to low-intensity exercise. In addition, it has been suggested that acute exercise leads to monocytosis irrespectively of intensity^33^. We confirm this finding now also in cancer patients as we did not find any associations between the changes in monocyte counts and blood pressure, heart rate, rate pressure product, mean arterial pressure, or pedaling power. Many of the exercise studies performed with healthy individuals use moderate-high intensity sessions that last at least 20 min, usually ranging from 20 to 120 min^11,19,21^. Critics suggest that the length and intensity used in these studies are too much for the general population, let alone for cancer patients. In this study, the intensity of the acute exercise bout was fairly low, which might have contributed to all patients not reaching sufficient intensities to elicit epinephrine responses, and maybe therefore we did not see increase in all leukocyte subtypes, as has been seen in other studies in healthy individuals^13,19,29^. However, findings in healthy people can not be generalized for cancer patients, so we can not assume that the mobilization would be similar even if the exercise intensity was higher. This study, however, shows a potentially useful clinical finding in that a short bout of low-intensity, supine cycling is sufficient to mobilize cytotoxic CD8^+^ T cells and NK cells in breast cancer patients. Interestingly, we found that exercise-induced mobilization of CD8^+^ T cells was negatively correlated with estrogen receptor positivity and positively with HER2 positivity in breast cancer. We also found no association between immune cell mobilization and breast cancer grade in this study, suggesting that exercise is beneficial irrespective of disease stage, but the benefit may, on the other hand, vary depending on molecular subtype. In our previous studies, we have observed an association between BMI and leukocyte count, but surprisingly not in this study. In a study with lymphoma patients in same settings we found that BMI correlated positively with change in NK cell count and granulocyte proportion between rest and exercise and with baseline levels of CD14^+^CD16^+^ and CD14^−^CD16^+^ monocytes^34^. Moreover, high BMI is associated with higher leukocyte count in otherwise apparently healthy people with various degrees of overweight and obesity^35^.

It is hypothesized that acute mobilization and redistribution of immune cells in response to exercise is a key reason why regular exercise elicits anti-tumor effects in both preclinical studies and in humans^14,36^, and it was therefore the focus of this study. In preclinical studies, it has been reported that high level of epinephrine and myokines released during exercise and redistribution of cytotoxic T cells and NK cells inhibit cancer cell growth^14,37,38^. After a single bout of exercise, the mobilization of immune cells may not have an impact on cancer but when repeated over time, the effect may accumulate^39^. Thus, based on our results, only a 10-min daily exercise might benefit breast cancer patients. Our results show that some immune cell subsets are mobilized during and after acute exercise bout, however, based on this study, we cannot know whether these cells are able to identify or kill cancer cells in tissues harboring tumors. Previous studies have shown an increased homing of leukocytes to e.g. lung and bone marrow after exercise^40^, but also an increased tumor infiltration by both NK cells^14^ and CD8^+^ T cells in exercising animals^38^. However, a study with prostate cancer patients did not observe differences in NK cell infiltrates in tumor tissue between people who conducted an acute exercise a day before surgery compared to the control group, but circulating NK cell count immediately before surgery was associated with NK cell infiltrates^41^. In addition, there are various factors that influence the susceptibility of tumor cells to exercise-induced immune surveillance. For example, the extent to which mobilized and redistributed NK cells might interact with cancer cells is likely determined by MHC-1 expression on tumor cells and whether tumor cells are stressed or damaged, or whether the tumor cells express tumor antigens^42^. Similarly, T cells mobilized to the tumor by exercise might or might not interact with cancer cells because of various factors that govern immunoediting, as outlined in cancer immunogram^43^. Understanding the cancer-immune system interaction better might help to explain why physical activity is associated with higher risk of some cancers but other cancers such as breast cancer show reduced incidence in highly active people^44^.

Our study has certain limitations. No individual maximal fitness tests were performed, so we were unable to determine the same relative intensity of exercise stress for each patient. This may contribute to the heterogeneity of the study results obtained between patients. Secondly, acute exercise can alter plasma volume due to sweating and increased muscle volume due to shift in fluids, thus affecting the concentrations of immune cells in the blood. However, there was no visible sweating in any of the patients due to short duration and fairly low intensity of exercise. Further, if plasma volume had decreased during exercise, the change would be reflected in an increase in the numbers of all immune cells studied, and also at the 30-min post exercise time point as patients were not drinking any liquids between blood sample withdrawals. We acknowledge that more frequent blood sampling after the cessation of exercise could have provided us more information about the temporal patterns of different white blood cell populations^45^ before 30-min time point. However, even if for instance T cells had increased at later time point, such as at 3-, 5- or 10-min time point, we could document that they are not increased at 30-min time point, as is also the case with most of the white blood cell populations studied here. In addition, some leukocyte concentrations appear to be low^46^, which may be due to immunosuppression due to cancer, or applied analytical methods. However, within this study, the changes in the immune cell counts are comparable as all samples were treated similarly before their analyses. Finally, exercise stress could improve the functionality of immune cells, even if the concentrations do not change significantly in the blood. We did not measure cytokine levels, which could have provided more insight on the topic.

In the precent study, we examined the changes on immune cell counts in breast cancer patients after acute, short 10-min bicycle exercise. We found that this acute exercise increased the number of total leukocytes (CD45^+^), CD8^+^ T cell, CD19^+^ B cells, total NK cells, CD56^+^CD16^+^ NK cells, and intermediate (CD14^+^CD16^+^) monocytes. In addition, the proportions of NK cells, CD8^+^ T cells, and CD14^+^CD16^+^ monocytes were increased by the exercise. Our study suggests that the positive effect of exercise on cancer might be partly due to immune cell mobilization and lends support to use of exercise as a standard part of cancer treatments. However, future studies are needed to examine the clinical significance of the effect of exercise on immune function.

## Materials and methods

This study was conducted at the Turku PET Centre, Turku, Finland between September 2020, and April 2021. Patients voluntarily participated by signing an informed consent form after reviewing the study information sheet and hearing an explanation about the study from the investigators. Good clinical practice and the Declaration of Helsinki were followed. The study was approved by the Ethics Committee of the Hospital District of Southwestern Finland and is registered in the international register of clinical trials (Clinicaltrials.gov NCT04416087).

### Participants

20 female breast cancer patients who had not started any cancer treatments were recruited to this study from Turku University Hospital. Exclusion criteria were abnormal fatigue, anemia, or physical dysfunction due to disease. Mean age was 58 ± 11 years and mean body mass index (BMI) was 28 ± 5 kg/m^2^. 13 of the patients had a ductal carcinoma and seven of them had a lobular carcinoma. Two of the patients had a primary metastatic disease. Twelve of the patients had a locally advanced disease with axilla lymph node involvement. Two of the patients had a triple negative disease while two of them had a HER2 positive disease. The proliferation index was between 2 and 75%, mean being 25%^47^. When the study was conducted, the patients had not started any cancer treatments.

### Study design

Participants underwent one visit at the study laboratory before the study day during which they tested pedaling a supine bicycle ergometer. During that subjective testing a pedaling power for the actual study visit was determined according to each participant’s own perceived fitness. Thus, testing was started with minimal power production and was gradually increased until the participant was confident that she could cycle with the chosen power for 10 min without fatigue development. During that test it was also determined that heart rate was at least modestly increased from rest**,** typically above 100 bpm. Higher power production levels were also tested to make sure that the choice was correct, and participant could not anymore cycle for several minutes with the higher intensity to make sure they did not choose too low power production level for the actual test. Strong physical exertion, alcohol consumption, and caffeine were prohibited for at least 24 h prior the study day. In the study participants conducted a 10-min supine bicycle exercise with a specific supine cycle ergometer (Tunturi E30^R^, Hungary). Prior to starting the measurements, an intravenous catheter was inserted for repeated blood sampling. In total 30 mL peripheral vein blood samples were taken to EDTA-tubes (BD Biosciences, San Jose, USA) from each participant, first at rest before the acute exercise, second immediately after exercise (within 1–2 min) and third 30 min after exercise. Heart rate (Palmsat 2500, Nonin, Plymouth, USA) and blood pressure (Apteq AE701f., Rossmax Swiss GmbH, Berneck, Switzerland) were measured at rest and during exercise. Rate pressure product (RPP) was measured by multiplying systolic blood pressure by heart rate. Mean arterial pressure (MAP) was measured by doubling the diastolic blood pressure and adding the sum to the systolic blood pressure and dividing that sum by 3. Age predicted maximal heart rate was measured by subtracting age from 220. Participants’ sensing of strenuousness of the exercise was determined with Borg scale.

### Flow cytometry

For flow cytometry, participants’ blood samples were analyzed within 12 h of sampling. Fc Block (BD Biosciences, San Jose, USA) was added to 100 µL blood samples and incubated for 10 min at room temperature. The samples were stained with fluorophore-labeled CD monoclonal antibodies (BD Biosciences, San Jose, USA). The CD antibodies used were CD45-FITC (clone HI30), CD3-BV605 (clone UCHT1), CD4-BV421 (clone RPA-T4), CD8-BV786 (clone RPA-T8), CD16-PE (clone 3G8), CD19-PE-CY7 (clone HIB19), and CD56-APC (clone NCAM162) for panel 1 and CD45-FITC (clone HI30), CD3-BV605 (clone UCHT1), CD14-APC (clone M5E2), CD16-PE (clone 3G8), CD19-PE-CY7 (clone HIB19), and CD64-BV421 (clone 10.1) for panel 2. Samples were incubated for 20 min in the dark at room temperature. Red blood cells were lysed with 1X FACS lysing solution (BD Biosciences, San Jose, USA) and incubated for 10 min in the dark at room temperature. Samples were washed with PBS (Thermo Fisher Scientific, Waltham, USA). After centrifugation, the cells were resuspended in 300 µL of PBS and samples were pipetted into a flat bottom 96-well plate. As applied previously^34,48–52^, samples were analyzed by running 150 µL of each sample with a BD LSR Fortessa™ flow cytometer (BD Biosciences, San Jose, USA) on the same day using BD FACSDiVa v.8 program (BD Biosciences, San Jose, USA). All samples were analyzed by the same person under the same conditions.

### Statistical methods

Flow cytometry data was analysed with FlowJo (BD Biosciences, San Jose, USA). Since half of the original blood sample (100 µl) was ran through the flow cytometer after staining and resuspension in PBS, the acquired cell count was multiplied by 2 and divided by 100 to express the results as cells per µl blood. Repeated measurement ANOVA was used to test the changes in immune cell counts. When the main effect (time) was less than *p* < 0.05, statistical differences in time points were considered by Tukey-post-hoc test. The associations between immune cell mobilization (Δ = cell count (exercise) − cell count (baseline)) and age, BMI, disease status, and exercise intensity variables were examined by Pearson´s correlation. Significance was determined at *p* < 0.05. All statistical analyses were performed with Graphpad prism 8.0.

## Data Availability

Raw data is made available from corresponding author upon reasonable request.
